# Effects and Mechanism of lncRNA CRNDE on Sepsis-Induced Acute Kidney Injury

**DOI:** 10.1155/2020/8576234

**Published:** 2020-05-01

**Authors:** Shen Wu, Hengyi Qiu, Qiao Wang, Zheng Cao, Jianmin Wang

**Affiliations:** ^1^Department of Emergency, Shanghai Ninth People's Hospital School of Medicine, Shanghai Jiao Tong University, China 200011; ^2^Department of Emergency, Hainan Western Central Hospital, China 571700

## Abstract

**Objective:**

To investigate the effects of lncRNA CRNDE on sepsis-associated acute kidney injury in the human kidney 2 cell line and explore the potential mechanisms.

**Methods:**

HK-2 cells were treated with lipopolysaccharides to induce injuries. The expression of CRNDE and miR-146a in HK-2 cells were altered by a transient transfection assay. Cell apoptosis was detected by a flow cytometry assay, and the levels of inflammatory cytokines including TNF-*α*, IL-6, IL-8, and IL-1*β* were assessed by ELISA. Furthermore, western blot analysis was performed to detect the expression levels of TLR4/NF-*κ*B pathway-related proteins. And a luciferase reporter gene assay was used to verify if miR-146a was the target of CRNDE.

**Results:**

LPS treatment increased CRNDE expression in HK-2 cells. CRNDE overexpression enhanced cell injuries in HK-2 cells significantly increasing inflammatory cytokine levels, including TNF-*α*, IL-6, IL-8, and IL-1*β*, and cell apoptosis. In addition, CRNDE overexpression further activated the TLR4/NF-*κ*B pathways in HK-2 cells. Inversely, opposite results were observed in the miR-146a mimic treatment group, and the miR-146a inhibitor could reverse the protein expression changes of TLR4/NF-*κ*B in the si-CRNDE and LPS treatment group.

**Conclusion:**

This study demonstrated that CRNDE overexpression could activate the TLR4/NF-*κ*B signaling pathway by regulating miR-146a, which accelerated LPS-induced inflammation and apoptosis in HK-2 cells.

## 1. Introduction

Sepsis is a systemic inflammatory response syndrome caused by bacterial (e.g., *Staphylococcus aureus*), viral, or fungal infections, and it is the main cause of death in intensive care units (ICUs) [1]. Sepsis can lead to organ damage. More and more studies show that acute kidney injury (AKI) is a common and serious complication in ICUs with sepsis, accounting for more than 50% of the ICU AKI cases, and the mortality rate is very high [2]. Inflammatory reaction caused by cytokines produced by sepsis is the main cause of multiple organ system failure; however, little progress has been made in the treatment of sepsis. Therefore, it is necessary to find effective targets for the treatment of sepsis AKI.

Lipopolysaccharide (LPS) is a component of the cell wall of Gram-negative bacteria. It has been proven that LPS can induce apoptosis of renal tubular epithelial cells [3]. LPS has been widely used in the establishment of an acute renal injury model in vitro [4, 5]. In mice and other animal models, LPS can induce acute inflammation by activating nuclear factor-kappa B (NF-kappa B) while inducing AKI [6]. NF-kappa B is an important transcription factor downstream of the endotoxin signal transduction pathway. When AKI occurs, an endotoxin induces cytokine production, accompanied by the activation of the NF-kappa B signaling pathway, which leads to a series of cascade reactions of cytokines (such as tumor necrosis factor-alpha, interleukin-1, and interleukin-6), i.e., inflammation, resulting in cell death, necrosis, and other injuries. Therefore, inhibiting the activation of the NF-kappa B signaling pathway may prevent the damage of AKI in sepsis.

lncRNAs are a group of RNAs [7] that are longer than 200 nucleotides without a protein-coding ability. In recent years, it has been reported that long noncoding RNAs (lncRNAs) are involved in the occurrence and development of sepsis. Therefore, exploring the mechanism of these lncRNAs in sepsis may help to find a new direction for the treatment of sepsis [8, 9]. Colorectal neoplasia differential expression (CRNDE) was originally considered as an lncRNA in colorectal cancer, which plays an important role in the proliferation, migration, and invasion of colorectal cancer cells [10]. Subsequently, there was evidence that CRNDE expression increased in glioma and renal cell carcinoma [11]. In addition to cancer, CRNDE has been found to promote inflammatory response. For example, Zhu-Ge et al. established the model of pneumonia and lung injury in vitro by inducing human lung fibroblasts with LPS [12]. It was found that CRNDE overexpression could accelerate apoptosis and inflammation induced by LPS through the upregulation of FOXM1. In addition, Li et al. overexpressed CRNDE in astrocytes and found that CRNDE upregulated the expression levels of NF-kappa B and many cytokines, thus triggering inflammatory response [13]. However, the role of CRNDE in the pathogenesis of sepsis and sepsis-related AKI has not been fully elucidated. We predicted that miR-146a might be the target gene of CRNDE through the StarBase database. Some studies have found that microRNA-146a can be used as an inflammation inhibitor to protect sepsis mice from organ damage [14]; Ding et al. found that microRNA-146a inhibited the activation of the proinflammatory signal pathway of NF-kappa B and the expression of downstream transcription factors [15] in LPS-induced AKI rat and cell models, thus playing an anti-inflammatory role. Therefore, we hypothesize that lncRNA CRNDE can play a role in sepsis AKI through the role of miR-146a on NF-*κ*B expression level. We intend to explore the mechanism of establishing an in vitro sepsis AKI cell model by LPS treatment of the human renal tubular epithelial cells.

## 2. Materials and Methods

### 2.1. Cell Culture and Treatment

Human renal tubular epithelial cell HK-2 (ATCC) was cultured in DMEM high-sugar medium containing 10% fetal bovine serum under 37 degrees Celsius and 5% CO_2_. According to Shen et al. [16], HK-2 cells were treated with 1 *μ*g/ml lipopolysaccharide (Sigma-Aldrich) for 24 hours to induce an acute kidney injury cell model of sepsis in vitro.

### 2.2. qRT-PCR

Total RNA was extracted from cells by the TRIzol Reagent (Invitrogen). The Reverse Transcription Reagent Kit (Invitrogen) was used to reverse transcribe RNA into DNA, and the ABI Prism 5700 system (Applied Biosystems) was used for qRT-PCR. With HAPDH as the internal parameter, the relative expression of CRNDE was calculated by the 2 delta CT method. Primer sequences are as follows: CRNDE—forward: 5′-TGAAGGAAGGAAGTGGTGGTGCA-3′, reverse: 5′-TCCAGTGGC ATCCTACAAGA-3′ and GAPHD—forward: 5′-CGTCGTA TTGGATTTAGG-3′, reverse: 5′-GAGCTTGACTTAGCCTTG-3′.

### 2.3. Transfection

CRNDE expression plasmid, CRNDE siRNA, microRNA-146a mimics, and microRNA-146a inhibitor were transfected into HK-2 cells using the Lipofectamine 3000 reagent (Thermo Fisher Scientific). The full-length CRNDE sequence was inserted into the pcDNA3.1 (Invitrogen) vector to construct the pcDNA3.1-CRNDE overexpression plasmid (O/E); the sequence of CRNDE siRNA (si-CRNDE) was sense, GUGCUCGAGUUAAAUTT, anti-sense, AUUACCACGAGCAT [17].

### 2.4. Detection of Inflammatory Cytokines

The concentration of inflammatory cytokines (TNF-*α*, IL-6, IL-8, and IL-1*β*) in the supernatant of cell culture was determined by an ELISA kit (Shanghai Biotechnicians). The results were expressed in pg/ml.

### 2.5. Flow Cytometric Detection of Apoptosis

According to the instructions, the apoptotic activity was detected by the Annexin V-FITC/PI Cell Apoptosis Detection Kit (Beyotime) and flow cytometry.

### 2.6. Dual Luciferase Reporter Gene

HK-2 cells were inoculated in the culture plate and cultured for 24 h, and the luciferase reporter plasmid pmirGLO-CRNDE-wt (wild type) or pmirGLO-CRNDE-mut (mutated type) and mir-146a mimics were transfected into the cells. Luciferase activity was detected by the Double Luciferase Reporter Assay System (Promega).

### 2.7. Western Blot

Cell proteins were extracted with RIPA lysate, separated by SDS-PAGE gel electrophoresis, and transferred to a PVDF membrane for antibody incubation. Clinx Science Instruments were used to expose and collect images. Primary antibodies are anti-TLR4 (Abcam), NF-kappa B p65 (Cell Signaling Technology), phospho-NF-kappa B p65 (Cell Signaling Technology), and GAPDH (Abcam).

### 2.8. Statistical Analysis

SPSS 20.0 software was used for statistical analysis, and the data were expressed as mean ± standard deviation. *p* < 0.05 was considered statistically significant.

## 3. Results

### 3.1. LPS Upregulated CRNDE Expression in HK-2 Cells

**Figure 1:**
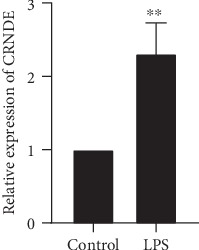
LPS-induced increase of lncRNA CRNDE expression in HK-2 cells. Compared with the control group, ^∗∗^*p* < 0.01.

First, we used LPS to stimulate HK-2 cells to construct the AKI cell model. qRT-PCR results showed that CRNDE expression was significantly increased after LPS stimulated HK-2 cells (Figure 1). These results suggest that LPS can upregulate CRNDE expression in HK-2 cells.

### 3.2. CRNDE Promotes Secretion of Inflammatory Cytokines

**Figure 2:**
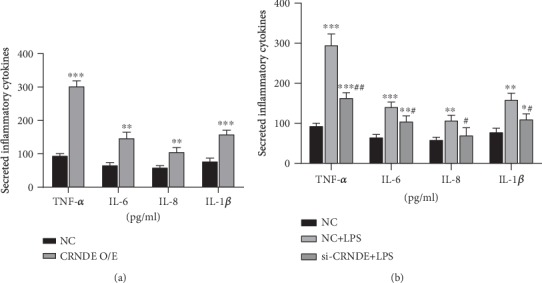
CRNDE-induced secretion of inflammatory cytokines by HK-2 cells. (a) Changes of cytokines in HK-2 cells that overexpressed CRNDE, compared with the control group, ^∗∗^*p* < 0.01 and ^∗∗∗^*p* < 0.001. (b) Changes in CRNDE inflammatory cytokine levels in LPS-treated cells, compared with the control group, ^∗^*p* < 0.05 and ^∗∗^*p* < 0.01, and compared with NC+LPS, ^#^*p* < 0.05 and ^##^*p* < 0.01.

After the overexpression of CRNDE in HK-2 cells, secretion of inflammatory cytokines TNF-*α*, IL-6, IL-8, and IL-1*β* increased significantly (Figure 2(a)). LPS also promoted the secretion of inflammatory cytokines. Silencing CRNDE in LPS-treated cells significantly reduced LPS-induced secretion of inflammatory cytokines (Figure 2(b)). These results suggest that CRNDE promotes the AKI process by promoting the secretion of inflammatory cytokines in HK-2 cells.

### 3.3. CRNDE Promotes Apoptosis of HK-2 Cells

**Figure 3:**
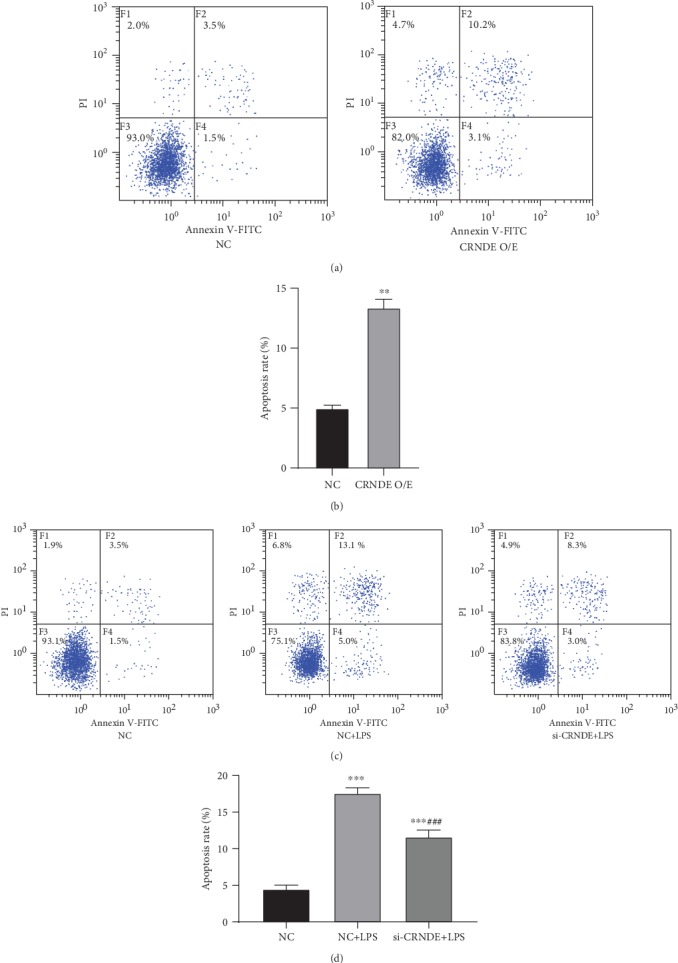
CRNDE promotes apoptosis in HK-2 cells. (a and b) Apoptosis of HK-2 cells that overexpressed CRNDE compared with the control group, ^∗∗^*p* < 0.01. (c and d) Apoptosis of silenced CRNDE cells treated with LPS: ^∗∗∗^*p* < 0.001 and ^###^*p* < 0.001 compared with NC+LPS in the control group.

After the overexpression of CRNDE in HK-2 cells, the apoptosis rate was significantly increased (Figures 3(a) and 3(b)). LPS treatment also promoted apoptosis. Silencing CRNDE in LPS-treated cells significantly reduced the LPS-induced increase in apoptosis (Figures 3(c) and 3(d)). These results suggest that CRNDE promotes the AKI process by promoting the apoptosis of HK-2 cells.

### 3.4. mir-146a Is the Target Gene of CRNDE

**Figure 4:**
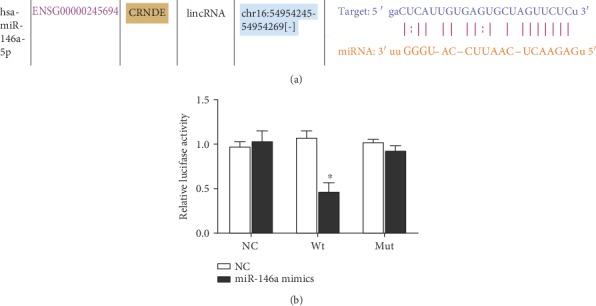
mir-146a was the target gene of CRNDE, ^∗^*p* < 0.05.

Through StarBase database prediction, we found that mir-146a may be the target gene of CRNDE (Figure 4(a)). The luciferase reporter gene results showed that overexpression of mir-146a in HK-2 cells could inhibit the luciferase activity of CRNDE-wt without affecting CRNDE-mut (Figure 4(b)). These results indicate that mir-146a is the target gene of CRNDE.

### 3.5. CRNDE Activates the TLR4/NF-*κ*B Pathway through mir-146a

**Figure 5:**
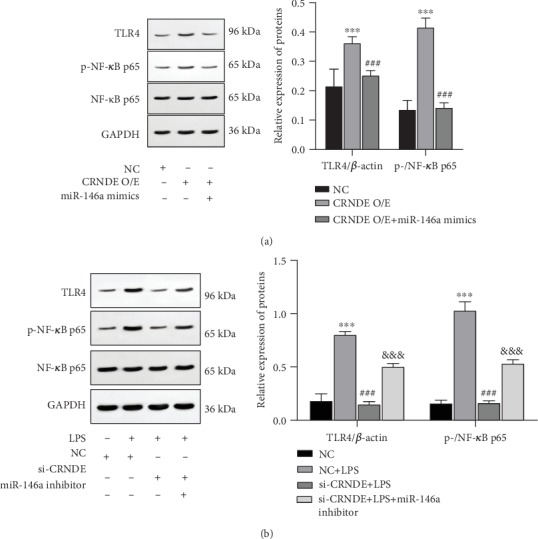
CRNDE induced the activation of the TLR4/NF-*κ*B pathway by mir-146a. Compared with the control group, ^∗∗∗^*p* < 0.001. Compared with NC+LPS, ^###^*p* < 0.001. Compared with si-CRNDE+LPS, ^&&&^*p* < 0.001.

Western blot results showed that the overexpression of CRNDE in HK-2 cells could activate the TLR4/NF-*κ*B pathway, while mir-146a could reverse the activation of the TLR4/NF-*κ*B pathway caused by CRNDE (Figure 5(a)). LPS could also activate the TLR4/NF-*κ*B pathway in HK-2 cells, and the silencing of CRNDE could significantly weaken the activation of the TLR4/NF-*κ*B pathway caused by LPS, while the mir-146a inhibitor could reverse the effect of CRNDE siRNA (Figure 5(b)). This result indicated that CRNDE activated the TLR4/NF-*κ*B pathway through mir-146a during AKI.

## 4. Discussion

In this study, it was found that in vitro LPS treatment of HK-2 cells could induce the increased expression of lncRNA CRNDE. Moreover, overexpression of CRNDE can activate the TLR4/NF-*κ*B pathway. In addition, luciferase reporter gene validation showed that mir-146a is the functional target of CRNDE, and mir-146a can reverse the activation of the TLR4/NF-*κ*B pathway caused by CRNDE. The above results suggest that lncRNA CRNDE plays a proinflammatory and proapoptotic role in LPS-induced septic AKI, and can activate the TLR4/NF-*κ*B signaling pathway by acting with mir-146a.

Recent studies have pointed out the direction for treating diseases by targeting lncRNAs [18]. CRNDE has been identified as an oncogene of various cancers, which is highly expressed in tumor cells and plays a role in regulating cell proliferation, migration, invasion, and apoptosis. For example, CRNDE is upregulated in colorectal cancer [10], gastric cancer [19], liver cancer [20], glioma [21], ovarian cancer [22], and other tissues. Increased CRNDE expression can promote proliferation, migration, and invasion of glioma stem cells and inhibit apoptosis by regulating mir-186 [21]. At the same time, CRNDE overexpression can enhance cell viability and promote the formation of cloning in gastric cancer cells by targeting mir-145 [19]. In addition, the study found that the TLR3/NF-*κ*B cytokine signaling pathway can be triggered by CRNDE and play a proinflammatory role in astrocytes [13, 23]. In the study on CRNDE and renal injury, Wang et al. established rat models of cellular renal injury and found that CRNDE alleviated the injury response through the miR-181a-5p/PPAR*α* pathway [24], while Sun et al. found the opposite result, that is, in a mouse model of renal injury, inhibition of CRNDE could block the activation of the TLR3/NF-*κ*B pathway and thus inhibit the renal injury response [25]. The results of this study are similar to those of Sun et al., that is, after LPS culture, CRNDE expression in HK-2 cells is significantly upregulated, and CRNDE expression promotes the inflammatory and apoptotic response of LPS-treated HK-2 cells, such as the increase of inflammatory cytokines TNF-*α*, IL-6, IL-8, and IL-1*β*. However, downregulating CRNDE can reduce the damage of LPS to HK-2 cells. These results suggest that CRNDE may play a key role in LPS-induced HK-2 cell inflammation. Current studies have found that CRNDE in renal injury can be positive or negative, which may be due to the heterogeneity of model animals, different experimental methods, and different focuses on pathways and functions. Therefore, it is suggested that we still need a large number of in-depth studies to clarify the role of CRNDE in renal injury.

The NF-*κ*B signaling pathway is essential in inflammatory response and plays a role in regulating cell activity, proliferation, differentiation, and tissue repair of inflammatory injury [25, 26]. Nonactivated NF-*κ*B exists in the cytoplasm, and when cells are stimulated by cytokines, infections, and toll-like receptors (TLRs) (e.g., TLR4), NF-*κ*B is rapidly phosphorylated and subsequently ubiquitinated and degraded. The degraded NF-*κ*B enters the nucleus and binds to the homologous gene sequence and induces the transcriptional expression of its target genes, such as inflammation-related genes [27, 28]. It is noteworthy that overexpression of CRNDE has been reported to increase the expression of NF-*κ*B and other cytokines, and lead to cellular inflammation [13]. In this study, it was found that the overexpression of CRNDE can activate the TLR4/NF-*κ*B signaling pathway, which may further explain the mechanism of CRNDE in LPS-induced inflammatory injury of HK-2 cells.

miRNAs are estimated to regulate more than half of the protein-coding genes in humans. In addition, miRNAs have been associated with a variety of human diseases [29]. For example, studies have found that miRNA-21 overexpression can play an antiapoptotic role in sepsis-induced AKI [30]. mir-146a is a typical multifunctional miRNA [31, 32]. Located on human chromosome 5, LOC285628 gene was first found in human acute single-cell leukemia cell line THP-1 [33]. The high expression of mir-146a may be related to the negative feedback regulation of the TLR and NF-*κ*B pathway under LPS induction [34]. In addition, studies have reported that mir-146a plays a role in LPS-induced injury as a target of multiple lncRNAs such as SNHG16 [35] and MALAT1 [36]. Through StarBase database prediction, this study found that mir-146a may be the target gene of CRNDE. The results were verified by luciferase reporter gene and consistent with previous prediction. In addition, we also found that mir-146a can reverse the activation of the TLR4/NF-*κ*B pathway caused by CRNDE, and the mir-146a inhibitor can reverse the effect of CRNDE siRNA, suggesting that CRNDE activates the TLR4/NF-*κ*B pathway through regulating mir-146a during the sepsis-induced AKI process. According to our previous studies, CRNDE may be an enhancer for the development of inflammatory response, but the deep mechanism of CRNDE's action still needs to be further studied.

## 5. Conclusion

In summary, this study showed that lncRNA CRNDE expression was upregulated in LPS-induced HK-2 cells, and CRNDE overexpression could promote the inflammatory and apoptotic response of LPS-induced HK-2 cells, and decreased CRNDE expression could weaken the LPS-induced injury to HK-2 cells. In addition, we also found that CRNDE can activate the TLR4/NF-*κ*B signaling pathway by regulating mir-146a. Our findings may provide new clues for understanding the pathogenesis of sepsis-induced AKI by CRNDE, and may provide a new target for the treatment of sepsis-induced AKI.

## Data Availability

All data generated or analyzed during this study are included in this published article.
